# Leeches and linchpins: a scoping review of seafood market intermediaries in small-scale fishery supply chains

**DOI:** 10.1007/s40152-026-00516-6

**Published:** 2026-10-19

**Authors:** Sharon K. Suri, Sahir Advani

**Affiliations:** 1 Department of Anthropology, Amsterdam Institute for Social Science Research, University of Amsterdam, Amsterdam, The Netherlands; 2 Department of Geography, Planning and International Development Studies, Amsterdam Institute for Social Science Research, University of Amsterdam, Amsterdam, The Netherlands; 3 Institute for the Oceans and Fisheries, University of British, Columbia, Canada; 4 School of Marine Sciences, University of Maine, Orono, USA

**Keywords:** Middlemen, Small-scale fisheries/SSF, Normative framing, Patron-client relationships, Traders, Power

## Abstract

Seafood market intermediaries, often termed *middlemen,* play a crucial yet contested role in connecting small-scale fisheries (SSF) to global seafood markets. While their influence has long been recognized, scholarly accounts have tended to frame these intermediaries as either exploitative actors who constrain fishers’ livelihoods or as essential actors who provide access to markets, finance, and social protection. This scoping review examines how recent literature (2019–2021) normatively frames seafood market intermediaries, and what these framings reveal about scholarly and policy discourses on SSF. Using a qualitative synthesis of peer-reviewed and grey literature, we identify predominant framings that cast these intermediaries as both leeches and linchpins, thereby reinforcing power asymmetries while also buffering fishers against economic and social risks. We discuss how these normative framings shape broader narratives that influence fisheries governance and policy, with implications for equitable outcomes. The paper also demonstrates the value of systematic scoping methods for synthesizing qualitative marine social science research and provides methodological reflections for small research teams. Recognizing the diverse, gendered, and socially embedded roles of intermediaries can inform more just and context-sensitive interventions in SSF seafood supply chains.

**Supplementary Information:**

The online version contains supplementary material available at https://doi.org/10.1007/s40152-026-00516-6.

## Introduction

Seafood market intermediaries (hereafter intermediaries) play a crucial role in connecting small-scale fisheries (SSF) with global seafood markets and consumers via seafood supply chains (hereafter, supply chains). Often lumped together under the label of middlemen, intermediaries occupy a variety of roles like collectors, cooperatives, distributors, patrons, traders, vendors, wholesalers, among other terms and through these roles influence sustainable, equitable, and wellbeing outcomes for fishing communities (Weeratunge et al. 2010). Intermediaries are highly heterogeneous in terms of gender, scale of operations, functions, and embeddedness in local economies, like other widely varied SSF actors (Short et al. 2021).

How these actors and their roles in SSF supply chains should be understood, however, has long been debated. Writing about Sri Lankan fisheries, Stirrat (1974), observed that, “[t]he normally accepted view is that [seafood] traders are parasites, living off and exploiting the impoverished peasants, the real producers of wealth in the economy, for whom the only hope is to form cooperatives and thus by-pass the traders in the distribution process” (pp. 189–190). Yet in the same study, Stirrat (1974) also documented the *good* side of intermediaries, noting how seafood traders connected socially with fishers by attending their marriages and funerals and provided bonuses, with the intent “to set up long-term *social* [emphasis in original] as well as economic relationships with the fishermen” (p. 204). This vacillation has persisted. We use the shorthand of *leeches and linchpins* to describe how the literature has cast intermediaries as actors who simultaneously take advantage of fishers but also support them. As leeches, echoing Stirrat’s “parasites”, they are framed as extracting value from fishers through unfair prices, debt, the obfuscation of market information, and inequities in fisheries governance (Fabinyi 2013; Johnson 2010; Purcell et al. 2017). Intermediaries have also been implicated in driving unsustainable fishing trends (Crona et al. 2010; Dacks et al. 2018; Kaplan-Hallam et al. 2017; Thyresson et al. 2013; Wamukota et al. 2014). As linchpins, they are framed as indispensable supply chain connectors, providing market access, finance, services, and social support that fishing livelihoods depend on. Scholarship is increasingly recognizing the pivotal role of intermediaries in enabling access to economic benefits from fishing livelihoods (Drury O’Neill et al. 2019a; Drury O’Neill and Crona 2017). Research on the gendered dimensions of intermediaries has progressively noted that women intermediaries are essential to the functioning of SSF, facilitating fish distribution, finance, the reproduction of labor, and social support in systems often characterized by struggle and negotiation (Bavinck 2018; Hapke 2001; Overå, 1993). Alternative forms of intermediaries, such as community-supported fisheries, also play a transformative role in ensuring equitable and sustainable fishing livelihoods (Cumming et al. 2020; Stoll et al. 2019). More than merely positive or negative labels, these framings carry competing claims about whether intermediaries are expendable or essential. Yet the emerging literature on justice in small-scale fisheries (Bennett et al. 2021; Cisneros-Montemayor et al. 2021; Cohen et al. 2019) only hints at the role intermediaries can play in fostering equity, food and nutrition security, and fisheries governance reform. As intermediaries continue to remain outside the purview of fisheries managers, policy makers, and academics, an understanding of how they are framed through the diverse roles they occupy must be highlighted and synthesized, which can inform research into the contexts that lead to either a leech or linchpin role in supply chain interventions.

A critical consideration of how intermediaries are represented and understood in the literature is necessary. The literature often carries normative framings, that is, implicit value judgements about how intermediaries *are* functioning or *ought* to function in supply chains. When considering these framings, we refer to how the literature assigns value or judgement to these actors, casting them as good, bad or somewhere in between. Such judgements reflect underlying scholarly assumptions about fairness, power, and efficiency in supply chains. These framings also sensitize researchers to certain aspects of social systems while obscuring others, thereby influencing our understanding of mechanisms and why they matter (Lindenberg and Steg 2007; Raza and Watts 2025). Examining these framings, then, goes beyond mapping scholarly discourse; it also reveals the implicit assumptions and resulting narratives shaping policy and interventions in SSF supply chains, such as Fair Trade or Blue Justice efforts (Cohen et al. 2019; Johnson 2006). These normative framings shape the narratives that influence whether intermediaries are seen as actors to be eliminated, circumvented, or supported within supply chains. Considering the full range of their influence, whether positive, negative, or nuanced, rather than a binary good or bad, can reveal a deeper understanding of their potential to transform supply chains towards sustainability and equity. For scientists and policy makers debating the future of seafood supply chains, this fuller view can inform more just and context-sensitive interventions.

We therefore ask: “*How does the literature normatively frame seafood market intermediaries in their roles connecting small-scale fishers and consumers?*” To answer this, we conducted a scoping review of the breadth of literature on intermediaries and the normative framings used to describe them. Within marine social sciences, impactful scoping and synthesis reviews have clarified definitions of SSF (Basurto et al. 2025; Smith and Basurto 2019), considered the focus on aquatic foods in food systems research (Simmance et al. 2022), and examined contributions of aquatic foods to food security and poverty reduction (Béné et al. 2016). However, few of these works provide transparent and practical advice on methodological considerations tailored to marine social scientists. Scoping reviews are valuable for marine social science because they are meant to map the conceptual breadth of a research field, while accommodating diverse epistemologies and methodological approaches. Unlike systematic reviews, which emphasize assessing the quality of research articles and statistical analysis of findings, scoping reviews prioritize transparency and reproducibility in searching, screening, and coding bodies of literature (Arksey and O’Malley 2005; Peters et al. 2015; Peterson et al. 2017). Methods to conduct scoping reviews of qualitative literature seem to be under-utilized despite their rigor and reproducibility (Andrews et al. 2025).

In order to allow for an appropriate level of depth and to serve as a methodological demonstration of how scoping reviews can be conducted by small review teams, this study focuses on a smaller, recent subset of the literature (2019–2021). The following section describes the methodology used to systematically scope the breadth of literature and our approach to extract and analyze normative framings of intermediaries from this subset. We then present the results on geographical and temporal trends and the predominant normative framings in research on intermediaries. We conclude with a discussion of both our findings on normative framings and methodological insights that scoping reviews offer to marine social science.

## Methods

We followed the recommended practice for reporting the methods of systematic scoping reviews, i.e., PRISMA-ScR (Tricco et al. 2018). We registered our final protocol a priori with the Open Science Framework on 11 June 2021 (Advani and Suri 2021). The aim of this scoping review was to synthesize recent literature on intermediaries, and through this process we demonstrated the value of scoping reviews in analyzing complex, qualitative research fields such as marine social science. Below, we provide detailed steps for how we conducted our scoping review, including decisions made to manage feasibility, maintain rigor, and facilitate collaboration using systematic review and qualitative coding software tools and adaptating to smaller teams.

### Defining study scope

This review aimed to determine the breadth of the literature on intermediaries and asked the specific question “*How does the recent literature (2019–2021) normatively frame seafood market intermediaries in their roles connecting SSF fishers and consumers?*” To answer this, we focused on peer-reviewed and grey literature that depicted intermediaries in this regard, particularly literature that described their roles, actions, and influence on supply chains.

Within our review, we defined intermediaries as actors in supply chains who distribute seafood between fishers and consumers. We limited our focus to supply chains involving seafood for human consumption and excluded studies related to solely non-edible uses (e.g., fishmeal, fish oil, pharmaceuticals) or actors in fisheries-allied sectors (e.g., boat builders, net menders) (see Online Resource 1). While we did not intentionally exclude other topics such as consumer-facing interactions or economic modeling, documents discussing these topics that did not also include normative framings were excluded.

Aquaculture and capture fisheries, collectively *aquatic food systems*, encompass an incredible amount of diversity in intensity, production systems, distribution channels, and more (Tacon and Metian 2013; Thilsted et al. 2016). For example, fisheries have spanned a range of sizes and intensities, from artisanal or SSF that use spears, single hook and lines, cast nets, etc., to industrial fisheries with large boats with fewer fishers and large corporations such as “keystone actors” (Johnson 2006; Osterblom et al. 2015; Pauly 2006; Smith and Basurto 2019). To actively include industrial fisheries would have entailed a largely distinct body of literature, which was neither our purpose nor feasible, thus we limited our scope to SSF. Similarly, while some aquaculture and mariculture supply chains overlap with capture fisheries supply chains, this would have entailed a prohibitively large set of articles, and as such, we limited our focus to small-scale capture fisheries. This conceptual narrowing was essential to manage the diversity of aquatic food systems literature while retaining a focus on SSF supply chain actors.

### Search strategy

We conducted literature searches in Scopus, Web of Science, and Google Scholar databases. Alongside peer-reviewed articles, we included grey literature such as policy briefs and reports that may sometimes be excluded from traditional reviews. Searches were restricted to title, abstract, and keyword fields. From Google Scholar, we screened the top 300 results by relevance (see Advani and Suri 2021 for additional details). As such, our search string focused on common terms for intermediaries and was limited to studies published in English and within the last 50 years (1971–2021). Due to the unknown quantity and quality of literature that would be captured, we intentionally cast a wide net with our search string. This strategy was also advantageous as it helped us measure the change in interest on the topic of intermediaries and include any influential literature published in earlier decades. The following Boolean search string was used within each database:

TITLE-ABS-KEY ((seafood OR fish* OR aquaculture OR mariculture) AND (trader* OR middlemen* OR vendor* OR fishworker* OR seller* OR buyer* OR intermediar* OR patron*)) AND PUBYEAR > 1970 AND (LIMIT-TO (LANGUAGE, "English"))

The final search was conducted on 14 June 2021 and returned 8,714 results. Results from each of the three databases were uploaded into Covidence, a web-based systematic review management software (Covidence 2025). Designed for systematic reviews, the Covidence platform is set up for teams to be able to progressively screen articles while checking if the different reviewers consistently make the same choices with regards to inclusion and exclusion. In this way, the bias of having a single reviewer assess each article is significantly reduced, resulting in a more replicable study.

### Screening and selection process

**Fig. 1:**
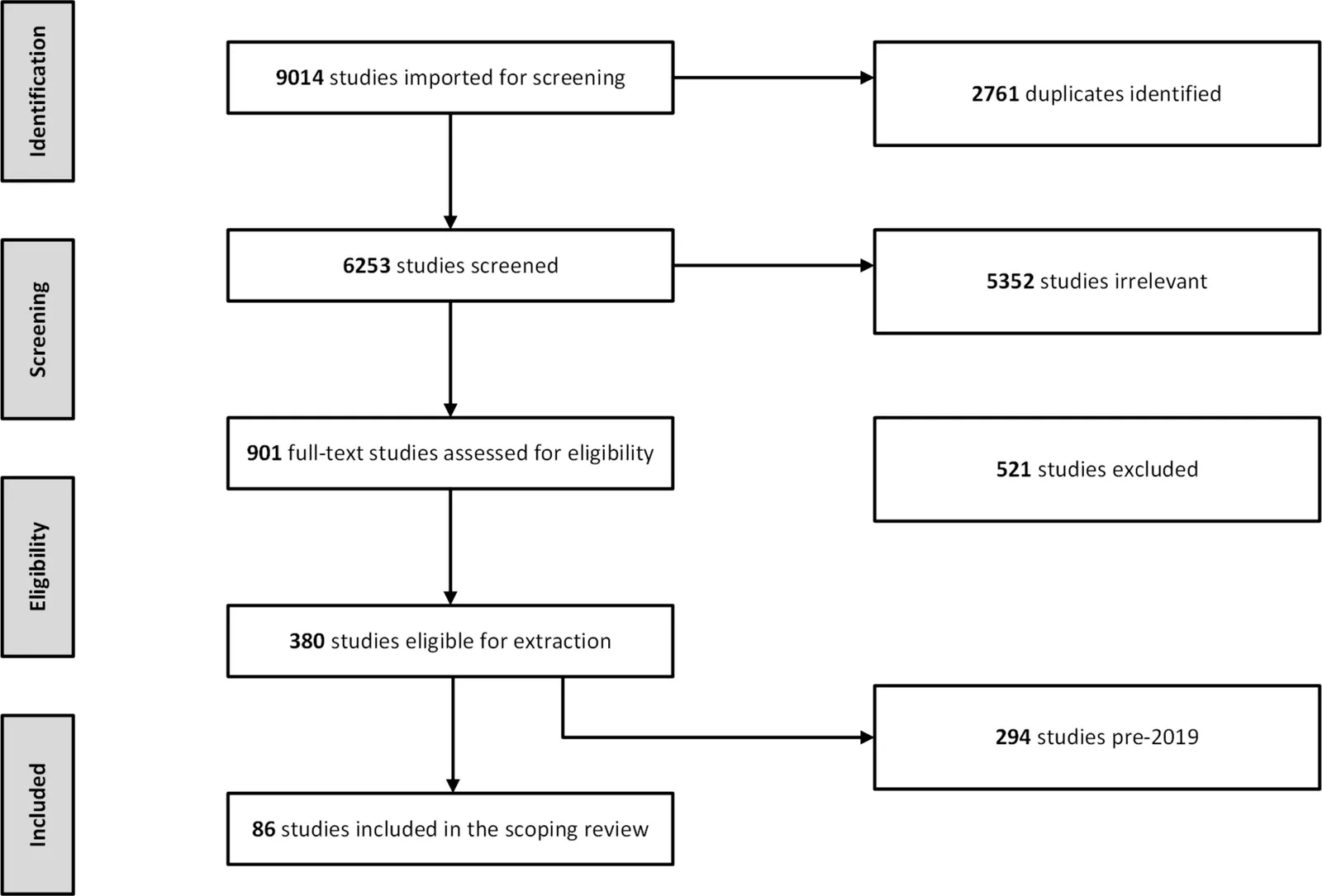
Prisma flowchart from Covidence, showing the number of documents excluded or included at each stage of screening

The screening process was conducted in four stages: deduplication, title and abstract screening, full-text screening, and selection of a recent subset of articles for data extraction. At each stage, Covidence was used to assign decisions, track reasons for exclusion, and maintain transparency in the review process (Fig. 1). In the first stage, deduplication, Covidence automatically detected 2,737 duplicates across the three databases and removed them, with an additional 24 duplicates being removed manually. The removal of 2,761 duplicates left 6,253 documents for initial screening of titles and abstracts. In reviewing the titles and abstracts, documents were excluded if they did not relate to SSF supply chains, or if they clearly related only to criteria outside the review’s scope, such as aquaculture, industrial fisheries, etc. This excluded 5,352 documents, leaving 901 documents to move to the next stage, full-text screening.

To screen the full texts, we first obtained digital copies of the documents and uploaded them to Covidence. Based on the title and abstract screening, we refined our list of inclusion and exclusion criteria to check as we each skimmed all 901 documents (see Online Resource 1). For every document we excluded, Covidence required reviewers to indicate a reason, which was then compared with the decision of the other reviewer to determine interrater reliability (Cohen’s Kappa = 0.98). After excluding 521 results, this left us with 380 documents for the in-depth full-text review (see Online Resource 2).

Using Covidence for the screening process facilitated surface-level analysis of the *full set* of 380 documents (from 1971–2021). Recognizing that an in-depth analysis of the full set of 380 documents was an excessive number for two reviewers to manage, we limited the full-text extraction to a smaller *subset* of documents. The subset could have been determined by randomized sampling of articles across the full 50-year time period, which then could have focused on the evolution of our understanding of intermediary roles in fish supply chains over time. However, recent discourse builds on past understandings of intermediaries while exerting stronger, more relevant influence on the broader policy work this review seeks to inform. We therefore prioritized a more recent analysis (as opposed to a randomized historical sample), including publications predating the COVID-19 pandemic and retaining at least 20% of the full set. The more manageable subset of 86 documents published between January 2019 and June 2021, and representing 22% of the total, allowed for the appropriate level of depth needed to engage in a qualitative scoping review, while also serving as a methodological demonstration of how scoping reviews can be conducted by small review teams.

### Data extraction

In this phase, we conducted an in-depth review of each of the 86 documents to extract all the information relevant to our research question. We also extracted basic summary data such as the countries of focus and any sub-national regions mentioned, if it covered marine or inland production systems, and more. Our established protocol (Advani and Suri 2021) was the starting point for determining the thematic concepts we read for; however, we intentionally built both closed and open-ended questions into the extraction form, creating additional space for previously unconsidered themes to emerge. This allowed us to iteratively revise the thematic concepts to incorporate those that arose during the full-text screening and previous steps. Table 1 details the thematic concepts we extracted and how we characterized them.

**Table 1:** Thematic areas of coding (left column) and their accompanying descriptions, detailing how they were characterized (right column)

Thematic concepts	Description
Normative framing	Examines how positively or negatively intermediaries have been framed in their relation to other actors in the chain. Articles were coded as framing intermediaries as: strongly or somewhat positive, neutral, strongly or somewhat negative, or both positive and negative. Then, the document text to support this selection was included.
Power and influence	Examines how explicitly power is discussed in relation to intermediaries. This included discussions of monopolies and oligopsonies. For example, was power explicitly/directly mentioned, referred to only indirectly, or not mentioned at all?
Price	Examines intermediaries’ influence/position in determining prices, if this was mentioned at all. For example, were they described as price makers or price takers?
What binds or links intermediaries with other actors	Examines the specific ways in which intermediaries are tied, linked or bound with other actors, if mentioned at all. For example, are intermediary-facing relationships linked via the following: credit or financial support, access to equipment, other services provided such as ice, kin or familiar relations, or by other means?
Gender and intersectionality	Examines if the document makes any references to gender or other diversity aspects as related to intermediaries. For example, does the document use sex-disaggregated data, mention gendered aspects, ethnicity, or religion?
Potential to leverage or transform	Examines the specific ways in which intermediaries might be considered as leverage points to create a more or less sustainable, just, or food secure system. Was this mentioned, and if so, how?

Similar to previous screening stages, each document was reviewed by both reviewers; however, rather than both reviewers examining and extracting all the relevant information, each document was extracted by one reviewer and then the extracted text and document was reviewed by the other to ensure consistency. The resulting extraction data in Covidence were then exported as a CSV file. This data was sparingly cleaned, to ensure consistency in terms and facilitate analysis of the spreadsheet in R in RStudio and Atlas.ti.

### Analysis in R and Atlas.ti

With the inclusion of both closed and open responses in the data extraction template, we were able to review both higher level summary information for the full set (N = 380), such as the distribution of documents over time and geographies, and the proportion of documents framing intermediaries as positive, negative, or both. We used open access software R (R Core Team 2025) in RStudio (Posit team 2025) primarily for quantitative analyses, while licensed software ATLAS.ti (2025) was used for thematically coding the extracted data. Publication metadata for the finalized documents included in our scoping review (N = 380) as well as the abstracts from the most recent subset (n = 86) were exported from Covidence and analyzed in RStudio to generate the figures presented in the results. For the data that were automatically collected by Covidence, such as publication year, we have reviewed the full 380 documents, whereas for data that were only collected through the extraction process, the analyses are based on the subset of the most recent literature (n = 86).

Using qualitative data analysis software ATLAS.ti, we imported the CSV file of extracted data, creating a separate document (in ATLAS.ti) for each paper. This allowed us to focus on solely the extracted sections of text, inductively coding to see what themes emerged. Inductive coding of the extracted text introduced additional nuance compared to a straightforward analysis of the content from the closed questions (Bernard et al. 2017). For example, several documents refer to intermediaries using language like “evil” or “deceptive,” framing them as villains in supply chains. For the closed question, this would be noted as strongly negative, but in ATLAS.ti, we were able to code for the nuance with this specific framing. The nuanced framings of intermediaries are presented and discussed in the sections below. We note that additional themes may have arisen within the full set (N = 380) (possibly including themes like consumers, auctions, economics), but below we only discuss the themes present in the subset (n = 86).

With the inclusion of both closed and open responses in the data extraction template, we were able to review both higher level summary information, such as the distribution of documents over time for the full set. Using R in RStudio, we were able to map the geographical distribution of documents. As there is a tendency for SSF research to overlook the contributions of inland SSF systems (Basurto et al. 2025), we share the percentages of documents based on marine and/or inland SSF systems below and other characteristics of intermediaries and SSF in our subset (n = 86) in the Supplementary Information (see Online Resource 1).

## Results

### Full set trends

**Fig. 2:**
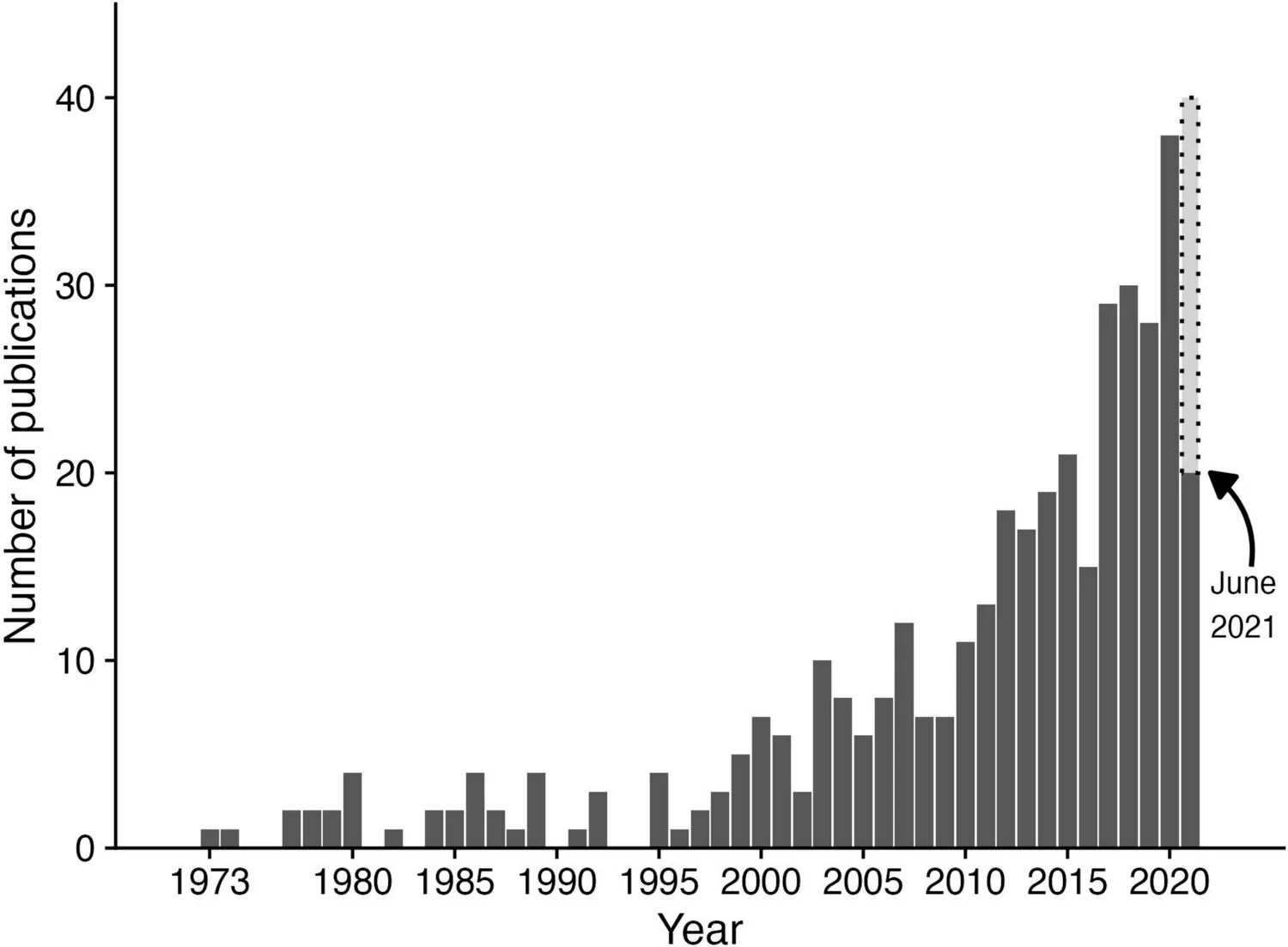
Number of publications from January 1971 to June 2021 (N = 380). As the search was conducted halfway through 2021, the light grey rectangle represents an estimate of the number of publications that would appear for the second half of 2021 (n = 40); this estimate is based on the number of documents published in the first half of the year. Note that this figure is based on the full 380 publications

Research on intermediaries evolved over the period considered in this scoping review. There was consistent growth in the amount of research (Fig. 2), but with a notable uptick since the mid-2000s. This may reflect increasing interest in this topic and/or an increased rate in scientific publishing more broadly. From 1971 through June 2021, the body of literature focusing on intermediaries consisted of 380 publications. As the search was conducted halfway through 2021, we doubled the number of results (see the lighter grey bar) to estimate the total number of citations for that year.

**Fig. 3:**
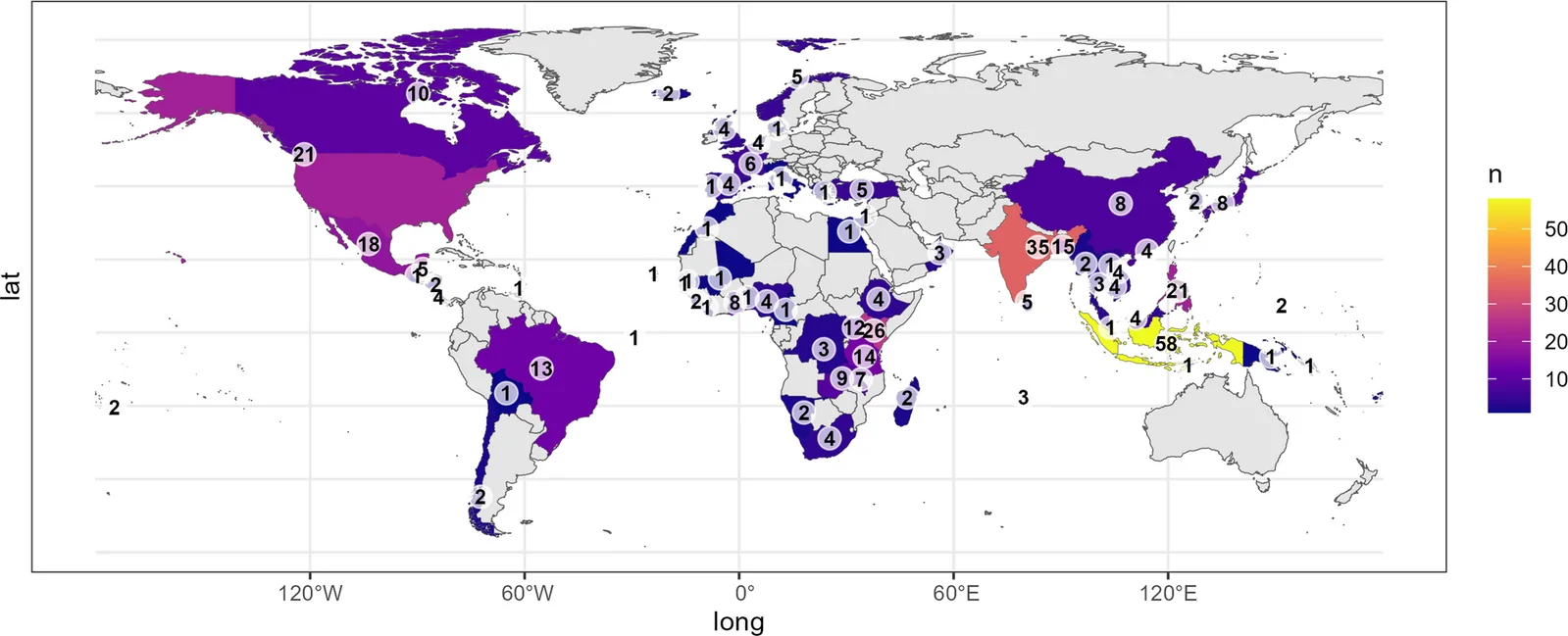
This is a heatmap showing which countries the documents (N = 380) focus on, with yellow representing more documents and dark purple representing fewer documents. The clear outlier here is Indonesia (in yellow) which was the geographical focus for 15% (n = 58) of the documents. The next most commonly mentioned country is India representing 9% (n = 5) of the documents

Our scoping review (N = 380) highlighted temporal and geographic trends in research on intermediaries (Fig. 3). The majority of documents focused on Asia, which is unsurprising given that 70% of aquatic animals are produced here (FAO 2024); of these, 58 documents were based in Indonesia. The next four most commonly mentioned countries were India (n = 35), Kenya (n = 27), the Philippines (n = 21), and the U.S. (n = 21). As Indonesia is the second largest capture fisheries producing nation (both industrial and SSF), we were unsurprised to see it as the most frequently targeted country (FAO 2024). Although China is the top capture fisheries producing nation globally (FAO 2024), it was a focus country for less than 3% of the documents. Potential reasons for this include that this capture fisheries statistic includes both SSF and industrial fisheries, whereas this review focused on SSF, and that this review only considered documents written in English.

**Fig. 4:**
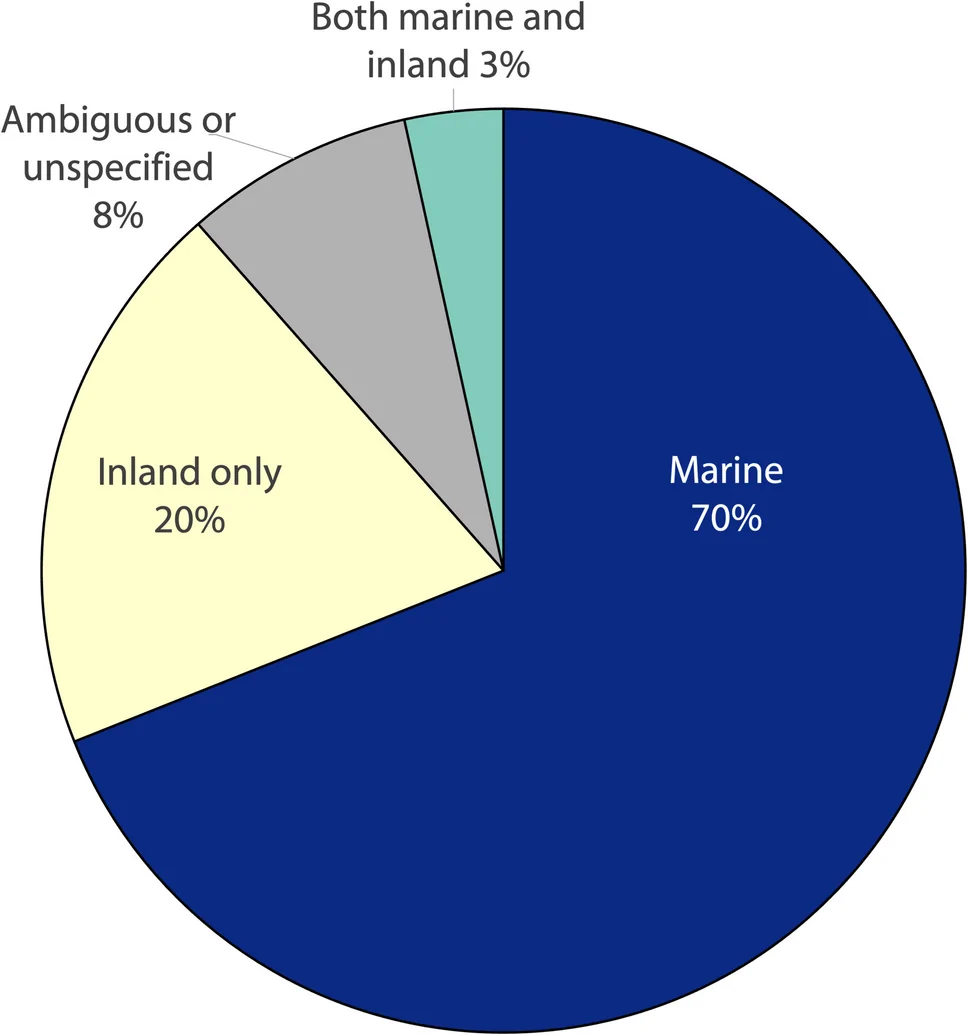
Proportion of documents (n = 86) referring to marine versus inland SSF systems. Documents were coded as referring to marine, inland, both marine and inland, or ambiguous or unspecified. The bulk of the documents looked at marine SSF systems (n = 60), while inland systems were one-fifth (n = 17) of the total, and less than one-tenth were ambiguous or unspecific about the systems referred to. Only a few (n = 3) documents referred to both marine and inland systems. Percentages may not add up precisely to the totals provided due to rounding

Spanning both marine and inland SSF, Fig. 4 shows the proportion of documents referring to the following SSF systems: marine (70%, n = 60), inland (20%, n = 17), both marine and inland (3%, n = 3), or ambiguous/unspecified (8%, n = 6).

### Normative framings in the subset

**Fig. 5:**
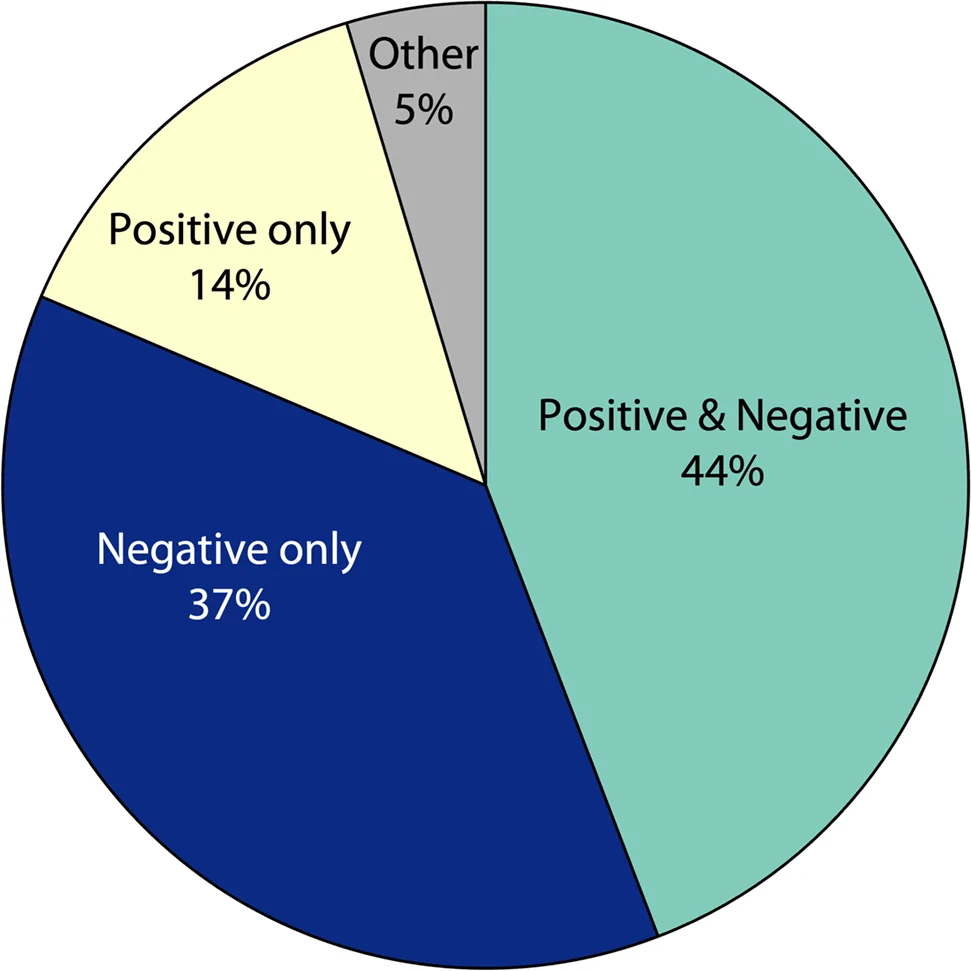
Proportion of documents (n = 86) framing seafood market intermediaries as positive only, negative only, both positive and negative, and other. This normative framing is in relation to their impacts on other actors in the supply chain and in the system overall. Percentages may not add up precisely to the totals provided due to rounding

The key focus of this scoping review is the normative framings of seafood market intermediaries across the literature; we documented if and how intermediaries are framed positively, negatively, or both. To determine this, we looked for depictions of how intermediaries interacted with other actors in the supply chain and how intermediaries affected the supply chain or the social-ecological environment around them. Of the 86 documents systematically coded, the majority (81%, n = 70) portrayed intermediaries in negative terms, with just under half (n = 32) discussing *only* negative aspects and a little over half (n = 38) combining negative with positive framings (Fig. 5). A smaller proportion (58%, n = 50) also documented positive roles of intermediaries, while only 12 documents (14%) described only positive framings. Four papers (5%) portrayed intermediaries as vulnerable or struggling actors without taking an explicitly positive or negative stance. While our search strategy included consumer-facing interactions and economic modeling, our subset of research did not include any articles examining this because documents discussing these topics that did not also contain normative framings were excluded. The sections listed below are organized from primarily negative (see Sects. "Dominance, power, and influence"–"Elimination or replacement as an improvement or goal") to primarily positive (see Sects. "Social protection and social insurance"–"Necessary for the supply chain") to more complex (see Sects. "Impacts on resource management"–"As vulnerable or struggling themselves"). As intermediaries’ interactions within supply chains are so nuanced, some sections include both positive and negative framings.

#### Dominance, power, and influence

Intermediaries’ dominance, power, and influence emerged as the most widely cited theme, appearing in 77% of reviewed documents (n = 66). Unlike other themes, it was often described as having both harmful and beneficial effects. This nuanced ambivalence reflects the bridging and central role intermediaries occupy in supply chains. Their ability to control access to global markets, shape prices, and govern relationships through influence in wider SSF systems, can both constrain and support fishers. Negative portrayals of dominance focused on aspects like: exploitation, market structures such as monopolies or oligopsonies, binding other actors (often fishers or suppliers) into exclusive business relationships, controlling prices, among other themes. Positive portrayals showed how intermediaries can leverage their dominance to the benefit of fishers and/or sustainability outcomes.

This power of large traders was strong enough to frame both fishers and small processors “at the *mercy* [emphasis added] of their buyers” (Visser, 2015, p.16, as cited in Duggan et al. 2020, p. 203; see also Adetoyinbo and Otter 2021; Kinseng 2020) Both Aswathy et al. (2020) and Hadi et al. (2020) positioned fishers and consumers as bearing the *brunt* or *burden* of big traders’ monopolies. Similarly, Kaminski et al. (2020) explored the gendered dimensions to this with female processors as “bear[ing] the brunt of losses compared to” (p. 16) male processors. Others also spoke to the gendered dimensions of these power imbalances, “which perpetuate the relationships of male dominance and female subordination” (Medard et al. 2019, p. 324). Speaking about the Nigerian context, Nwabeze et al. (2019) positioned middlemen as “enjoying unearned income depriving the actual fishermen of the benefit of their labour and production” (p. 55), a sentiment shared across many regions. Thus, “the real economic benefits from fishing are often skewed in the hands of a few intermediaries in the value chain” (N. Aswathy et al. 2020, p. 123).

The bargaining power of intermediaries over others in the supply chain was frequently raised across local to global levels. Elsler et al. (2021) described how traders, “as gatekeepers to global markets … create dependencies and reduce fishers’ ability to negotiate competitive prices” (p. 35). This “heavy market power of middlemen” directly impacts fishers’ economic dependence on intermediaries and is “one of the main reasons why the incomes of small-scale fishers are worsening” and can stymie attempts at more equitable endeavors such as cooperatives (Ertör-Akyazi 2020, p. 54; see also Ivars and Venot 2020). This market power was often gained through the provision of finance and/or equipment with “middlemen in a powerful position because they can dictate prices and apply leverage by withholding fuel, fishing nets and other gear, and access to markets and services” (Busilacchi et al. 2022, p. 63), leaving fishers “trapped in this system,” with few options to leave (Darwis and Zulkarnain 2020, p. 3). Through the provision of credit, “middlemen wielded higher control in the value chain, which was reflected in their bargaining power” (Jueseah et al. 2020, p. 6). Due to fishers’ (and even some cooperatives’) dependence on capital, wealthier supply chain actors have been able to “retain control over fishery resources” (Ivars and Venot 2020, p. 54), typically “creating downward pressure on ex-vessel prices” (Peña-Torres et al. 2019, p. 375). As described by Yagi (2019), “[f]ishers have no choice but to accept risk-averse attitudes of distributors or retailors [sic], because fishers are in a weaker position” (p. 217).

This dominance and power of intermediaries over others in the supply chain was at times presented as more nuanced relationships where fishers’ and boat owners’ control over scarce fish supply granted them “a degree of power over fish agents and power to achieve better prices and credit conditions” (Nunan et al. 2020, p. 251). When financing and/or fishing equipment has been provided in advance, such as in patron-client relations, intermediaries must place their trust in fishers to both maintain these assets and to deliver the entire catch to these same intermediaries providing these services, thus indicating another element of fishers’ power over intermediaries (Nunan et al. 2020). Some discussions of patron-client relations note that fishers accept patrons’ control as the financial and social help provided and the risks absorbed are worth the trade-offs of intermediaries’ greater power in the relationship, as discussed in the social protection section (Hamzah and Nurdin 2021; Jimenez et al. 2020; Kinseng 2020; McWilliam et al. 2021; see Sect. "Social protection and social insurance").

Intermediaries were also seen to wield their influence in more positive ways such as: obtaining necessary documentation like permits (Doddema et al. 2020a), ensuring upstream compliance with environmental regulation requirements, and actively supporting fishing communities (Busilacchi et al. 2022). Interestingly, certain intermediary actors (e.g., marketing cooperatives) used their power to buffer fishers against other intermediary actors (e.g., more traditional middlemen), as seen with the Matsyafed cooperative in India (Parappurathu et al. 2019). While not all examples have been successful, cooperatives have often been suggested as an alternative market intermediary to counter the dominance of, or eliminate, existing middlemen (see Sect. "Elimination or replacement as an improvement or goal").

#### Exploitation and manipulation

Roughly a quarter of the documents (n = 22) discussed the existence of, or potential for, intermediaries exploiting other actors in the supply chain. This predominantly negative framing was most commonly described as intermediaries exploiting fishers, but in some instances, women traders were the ones being exploited by other intermediaries. For example, in Tunisian clam fisheries, “women are exploited by intermediaries who impose low prices and sell these [clams] at higher prices at the markets in Europe” (Cele 2020, p. 144). In Zambia, Kaminski et al. (2020) documented how "'outside' traders" exploit women’s family responsibilities and the perishable fish they sell by deliberately “waiting until the end of the day to offer lower prices for the fish, pointing to the lack of freshness as justification” (p. 11). Similarly, Medard et al. (2019) described how hierarchical trading networks around Lake Victoria “create an exploitative network in which powerful middlemen control the access of trade for women from the Democratic Republic of Congo (DRC) and marginalizes [sic] the Tanzanian women” (p. 321), at times leading women to engage in transactional sexual relationships to secure access to fish.

Several studies explicitly linked exploitation to the broader pattern of dominant power held by intermediaries within supply chains (Aida et al. 2020; Kinseng 2020). First-hand accounts from fishers vividly described this dynamic. One fisher in Indonesia shared a proverb that contrasts the lives of patrons (*punggawa*) with fisher laborers (*sawi*), “punggawa: relax, be extravagant, but very rich; sawi: bone breaking, bone fracture” (Kinseng 2020, p. 66). In Turkey, another fisher did not mince words saying, “[t]he middlemen are exploiting the fishers” (Ertör-Akyazi 2020, p. 54). The majority of these depictions specifically linked exploitation to finance, both the provision of credit to finance fishing operations and the control over sale prices. In these instances, intermediaries were often labeled as ‘middlemen’ or ‘patrons’, emphasizing their role in provisioning capital and/or fishing equipment such as boats. As Thủy et al. (2019) detailed, “[m]iddlemen may exploit their power and take most of the rent to the detriment of fishermen. A welfare economic optimum will not be achieved, since in this scenario the rent mainly accrues to a few middlemen” (p. 451). McWilliam et al. (2021) emphasized the structural inequality of these relationships as, “[t]he consequences of these long-term interactions between patrons and clients is the emergence of structural inequalities and marked differences in levels of household prosperity in the surveyed Sama Bajo settlements. In other words, the punggawa patron is both an architect of debt and economic vulnerability, even exploitation” (p. 145).

Some studies went further in detailing the intentions of intermediaries in creating exploitative situations, for example through “corrupted leaders colluding with buyers to exploit fishermen on the low landing price” in Tanzania (D. B. Sambuo et al. 2021, p. 356) or in India where interest rates on loans can be exorbitant and “lender-auctioneers indulge in several fraudulent malpractices that essentially lead to under-pricing of the transacted fish” (Parappurathu et al. 2019, p. 109). Intermediaries’ exploitative interest rates on credit and “forced purchase price[s]” reinforce cycles of debt when fishers aren’t able to repay loans to intermediaries (Kinseng 2020, p. 66). Reflecting more broadly on these relationships in supply chains globally, Pomeroy et al. (2020) noted that “​​[t]he terms of finance are often disadvantageous for fishers. The lender can charge high rates of interest, without disclosing the true cost of the debt, and the relationship can be exploitive of the fisher” (p. 3). They went on to argue that "[t]he level of indebtedness is one of the underlying causes of their sustained poverty and a main obstacle in escaping from it” (Pomeroy et al. 2020, p. 3), thus directly linking exploitative practices to the persistence of structural poverty in fishing communities. This connection between exploitative financial arrangements and persistent poverty was a common finding across the literature and points to another major theme, the inequitable and asymmetrical nature of intermediary-fisher relationships.

#### As bottlenecks

Just under half of the documents (n = 36) characterized relationships with intermediaries as asymmetrical and as monopolies or oligopsonies, which can function as bottlenecks in the supply chain between fishers and consumers. These characteristics have also been linked to other themes such as “dominance and exploitation” (Aida et al. 2020, p. 76), highlighting that “power relationships are asymmetrical, with small-scale fishers holding the weakest position,” (Jimenez et al. 2019, p. 6), which “can lead to exploitative social relations, whereby traders uses [sic] their position to drive down the purchase price of fish products” (Jimenez et al. 2020, p. 2). Comparing fishers in asymmetrical versus symmetrical relationships, Darwis and Zulkarnain (2020) noted that the former were “trapped” (p. 4) in these relationships, while the latter were more successful in exiting poverty.

Other documents emphasized the vertical or hierarchical aspect of this asymmetry. In Chile, the "[c]entolla fish chain is characterized by a very hierarchical configuration” (Nahuelhual et al. 2020, p. 112), and Peña-Torres et al. (2019) detailed the “strong vertical control” (p. 364) of importers over hake processors. In local supply chains in Nigeria, Adetoyinbo and Otter (2021) found that “traders were ranked as the overall lead actor” (p. 194), a finding echoed by many documents. This configuration facilitated “an extra layer of rent-capture within a system that was already vertically structured" (Ivars and Venot 2020, p. 525). Several documents specifically noted the “captive type of coordination among traders and fishers” in these supply chains (Coronado et al. 2020, p. 5; see also Adetoyinbo and Otter 2021; Jueseah et al. 2020).

The context of monopolies and oligopsonies was raised often in the subset, with some describing this structure as an expected characteristic of the system, while others positioned intermediaries as actively enforcing these market structures. According to Muis et al. (2020), “middlemen control the market, therefore, they monopolize trade routes, and create dependency” (pp. 2224–2225). Khan et al., (2019) described “a marketing system which is completely monopolised by middlemen” (p. 639), and similarly Sambuo et al. (2020) repeatedly referred to “monopolistic middlemen” (pp. 1826, 1833), particularly in relation to fish pricing. Both Aswathy et al. (2020) and Hadi et al. (2020) highlighted the monopolies of large traders as bearing down on fishers and consumers. Thủy et al. (2019) discussed how “credit arrangements and reciprocal agreements” add “to the oligopsonistic power of the middlemen” (p. 448).

#### Binding or limiting the options of others

Half of the documents (n = 43) highlighted the binding nature of intermediaries’ relationships with other actors, typically upstream fishers, thus limiting the options or choices of those other actors. This was most commonly done through controlling access to capital where fishers “are often forced to borrow money and other daily necessities from traders or collectors” (Aida et al. 2020, p. 76) leading to “indentured work forces” (Vandenberg et al. 2021, p. 76) where “fishermen are bound by debt without knowing when they can pay it off” (Hamzah and Nurdin 2021, p. 6). This was sometimes compared to imprisonment; for instance “the moneylender and middleman’s snares that have been shackling them” (Anwar et al. 2019, p. 308), with one Indonesian fisher describing their situation as, “[m]y neck is tied up… Unless a fisherman pays his debt he will be bound to punggawa [financier middlemen]” (Kinseng et al. 2019, p. 64).

Beyond finance, ties between intermediaries and other actors, most typically upstream fishers, were also maintained through the provision of equipment and services. In exchange for fishing gear such as boats and nets and consumables such as fuel and ice (sometimes at marked-up prices), fishers were required to sell their harvest directly to, and at a price set by, intermediaries such as processors (González-Mon et al. 2019; MacKeracher et al. 2019; Noegroho et al. 2020; Nwabeze et al. 2019). Where permits were required for fishing, these were often secured by intermediaries who provide or rent them to fishers in exchange for priority access to the harvest, forming another binding tie between them (Basurto et al. 2020; Doddema et al. 2020a; Elsler et al. 2021; Siegelman et al. 2019). The bureaucracy and lack of infrastructure in which many supply chains operate mean that intermediaries are often the only means for fishers to access these equipment and services. Though sometimes a source of conflict, the provision of these functions to strengthen the loyalty between fishers and intermediaries (Doddema et al. 2020a; Jimenez et al. 2019).

The benefit of this indebtedness and linkage to intermediaries is generally the supply chain control this affords them, thus enabling them to control prices and ensure a steady supply of seafood, guaranteeing “exclusive access to purchasing landings” (Bevilacqua et al. 2019, p. 329; see also Doria et al. 2021; Purnomo et al. 2020; Wardono et al. 2020). Some fishers have described this binding relationship as positive in that it is an accessible source of credit in an otherwise risky business (Pomeroy et al. 2020), assures them of a buyer for all their seafood (Saputra 2020), and can function as a kind of social protection (Hamzah and Nurdin 2021; McWilliam et al. 2021; Nurlaili et al. 2019). These ties are “also a major factor that restricts client choices to turn to alternative non-fishing income-generating activities” (McWilliam et al. 2021, p. 145) and that “constrain fisher’s market related behavior” (Drury O’Neill et al. 2019b, p. 12).

#### Contributing to the inequity or inequality of others

Intermediaries’ contribution to greater inequality was discussed in 40% (n = 35) of the documents and was often described as persistent and systemic (Aida et al. 2020; McWilliam et al. 2021), with “the engrained perception of patron-client relationships as the default system, despite its inequity” (Drury O’Neill et al. 2019a, p. 7). Inequality was linked to both dominance and exploitation by intermediaries and the structural inequity of the patron-client system frequently underpinning relations between intermediaries and fishers. This was also closely linked to the binding nature and asymmetrical form of these relations, where “fishers disproportionately shouldered the risk compared to middlemen” (Kimani et al. 2020b, p. 7) and are often the “most marginalized community” (Bhuiyan et al. 2021, p. 331). Even the social support provided by patrons “inhibits fishers from achieving substantial financial autonomy,” and “has been criticised as a mechanism that creates a poverty trap,” and can thus “impede local fishers from improving their food security” (Vandenberg et al. 2021, p. 75).

Beyond patron-client relations, similar inequities were reported in other intermediary contexts, with fishers at times receiving half the revenue of distributors (Bevilacqua et al. 2019) and sometimes earning less than the lowest minimum wages (Fitrianggraeni 2019). These low incomes combined with high levels of indebtedness created a system of inescapable structural poverty for fishers (Darwis and Zulkarnain 2020; Kinseng et al. 2019; Nurlaili et al. 2019; Pomeroy et al. 2020). As noted by Nahuelhual et al.(2020), “[t]hese inequalities keep the system working, acting as a motive for illegal practices” (p. 112).

#### Elimination or replacement as an improvement or goal

Eliminating intermediaries was brought up in over a fifth (n = 19) of the documents. Intermediaries (often termed as middlemen in these cases) were framed as the source of problems in the supply chain and in some cases “unnecessary” (Hadi et al. 2020, p. 620). In some instances, the absence of intermediaries (typically those providing finance) was suggested as the reason why supply chains were functioning well for fishers. “The existence of a simple and direct marketing pathway in this value chain is owed to the absence of middlemen who disproportionately capture wealth among value chain actors” (Uba et al. 2020, p. 115). The elimination of these intermediaries was most frequently suggested as a strategy that would benefit fishers, typically through increased profits and reduced exploitation. Fishers “believed that the elimination of commercial intermediaries or at least a better price paid for fish could generate more profit and consequently a faster return on invested capital” (Inomata et al. 2020, p. 7489).

Other documents went further in the practicality of eliminating intermediaries by suggesting potential alternatives. As per Ertör-Akyazi (2020), one small-scale fisher in Turkey shared, “[t]he middlemen are exploiting the fishers. This system [of middlemen] needs to be abolished” (p. 54), proposing that fishers instead work with cooperatives. In Liberia, Jueseah et al. (2020) suggested that microloans from the national government could, “break [fishers’] financial dependencies on dominant middlemen” (p. 9). In Indonesia, Islamic microfinance, via ‘Salam’ contracts, “identifies the problems, educates family financial management, and fulfills the members’ financial needs according to the season cycle, in order to make them not get into debt with moneylenders and wholesalers” (Anwar et al. 2019, p. 308). In Kenya and Indonesia, government support was suggested for subsidizing equipment, overseeing marketing spaces such as auction houses, and creating price indexes that would allow fishers to “wean off dependence on middlemen” (Kimani et al. 2020a, p. 5; see also Widiastuti and Kamaluddin 2019).

Many documents (n = 9) mentioning cooperatives did so briefly as a potential recommendation to improve the supply chain, typically to position fishers more equitably (Fitrianggraeni 2019; Jimenez et al. 2020; D. Sambuo et al. 2020; Uba et al. 2020), reduce the dependency of fishers on middlemen (Nwabeze et al. 2019), or to provide an alternative to or outright eliminate middlemen or other intermediaries (Hamzah and Nurdin 2021; Jimenez et al. 2019; Kimani et al. 2020a; Kinseng 2020). Beyond the potential recommendations, these documents rarely provided evidence to support this in practice, while there were numerous cases where alternatives had failed in this endeavor. In India, the primary cooperative (PFCS) “failed in eliminating middlemen from the Chilika fishery” due to “the absolute absence of fish marketing by PFCS, a dominance of commission agents and weak subleasing practices” (Baral 2019, p. 631). Similarly in South Africa, “four separate attempts to brand linefish and cut out the middlemen were made: two by businessmen and two by local linefishers. Each attempt failed" (Duggan et al. 2020, p. 196). Of the documents that more substantially mention cooperatives (n = 9), only one case from Kerala, India detailed how cooperatives have been a positive force for fishers. “In fact, many developments and reforms in the domain of fishery credit delivery, fish marketing and social safety net coverage for fishers in the state have been achieved with the intermediation of cooperatives" (Parappurathu et al. 2019, p. 102). According to Basurto et al. (2020), “often gradients can be found in between” (p. 6) patron-client relations and cooperatives; however, cooperatives are more likely to equitably engage fishers. Coronado et al. (2020) noted that relationships with coops can range, “from commercial to captive, depending on the assets the cooperative owns and the historical relationships” (p. 5). Most of these articles (n = 5) instead discussed cooperatives in terms of how they failed to achieve their goals in practice, which often included replacing or eliminating middlemen (Baral 2019; Ertör-Akyazi 2020); this was at times due to threats from other actors (Getahun et al. 2020), being overly restrictive of member fishers (Kamaylo et al. 2021), or outright corruption (Pedroza-Gutiérrez and López-Rocha 2021).

#### Social protection and social insurance

Relations between intermediaries and upstream actors often included elements of social protection or social insurance, where intermediaries provided assistance to fishers beyond material requirements of seafood production, a theme addressed by a fifth of the documents (n = 19). For fishers, “the patron-client relationship is a guarantee of their social security" (Aida et al. 2020, p. 75). Going beyond finance for fishing inputs or equipment, patrons also provided “cash for personal loans (e.g., payment of corner-store bills, health emergencies, etc.)” (Basurto et al. 2020, p. 6). In Lake Victoria, these “social obligations” meant that boat owners “must try to meet their demands like providing food, providing treatment when one is sick, and taking care of fishers’ needs” (Nunan et al. 2020, p. 250). In some geographically distant places, services provided by middlemen “supplement the limited government services and allow South Fly villagers to access Indonesian education and health services in Merauke” (Busilacchi et al. 2022, p. 61). As described by a woman in one of these villages, “when they come from Indonesia, they live with us in our home, we accommodate them, and what they bring they share with us. They become part of the family ... they are good people. They are very open, and they also help us with some of the situations” (Busilacchi et al. 2022, p. 61). Despite the asymmetric relationships they promote (González-Mon et al. 2019), in patron-client relations clients saw their patrons as a “social safety net in circumstances where, historically there have been few alternative options” (McWilliam et al. 2021, p. 145). Other documents also flagged the trade-offs that frequently accompany these social practices, noting consequences for fishers’ debt, bargaining power, and even implementation of traceability/certification standards (Doddema et al. 2020b; Jimenez et al. 2020; Kinseng 2020).

#### Improving the supply chain or situation of other actors

One-third (n = 30) of the documents recognized the positive contributions of intermediaries to other actors in the supply chain or its overall resilience. Though “the degree of benefit was different” between patrons and clients, even these systems can “encourage [the] vertical mobility of fishermen” (Hamzah and Nurdin 2021, p. 6). Intermediary-related benefits included enhanced food security, increased adaptive capacity, improved access to finance, and assistance in overcoming bureaucratic hurdles. In the USA, the community supported fishery Fishadelphia’s aims explicitly targeted “increasing access among low-income consumers to high-quality protein and expanding domestic markets for seafood harvesters” (Cumming et al. 2020, p. 71). In East Africa, D’agata et al. (2020) found that, along with higher levels of education, “the presence of market traders (middlemen) had a positive relationship” (p. 56) on social adaptive capacity.

Fishers often preferred the financial terms of traders because of their willingness to forgo collateral and to accept payment as a portion of the harvest; in the event that fishers defaulted, their debt could be paid through the return or sale of their equipment, allowing them, “to take an honorable exit from the debts involved” (Parappurathu et al. 2019, p. 109). Traders also typically offered simplified financial terms, as compared to banks, which often considered fishing an “untrusted” business that was ineligible for finance (Wardono et al. 2020, p. 4). As an intermediary between fishers and wholesalers, Islamic microfinance institutions (Islamic MFIs) limited the ability of price manipulation, thus improving the bargaining power of fishers (Anwar et al. 2019). Traders, exporters, and suppliers played a key role in facilitating access to fishing permits required for traceability standards for smaller vessels, thus enabling them to benefit from Fair Trade price premiums in Indonesia (Doddema et al. 2020a).

#### Necessary for the supply chain

Through a variety of means, in 29% (n = 25) of documents intermediaries were often recognized as necessary actors in supply chains. Their ability to facilitate fishing though equipment and capital was essential to fishers to be able to go to sea and fish. Without the financing and equipment provided by intermediaries, many fishers would be unable to go to sea and fish (Bevilacqua et al. 2019; Hamzah and Nurdin 2021; Kimani et al. 2020a; Vandenberg et al. 2021). Intermediaries often committed to buying the full catch of fishers, which otherwise might be difficult for local markets to absorb. “There are no indications that all fish produced by small-scale fishermen in both value chains could be sold at market price" (Jueseah et al. 2020, p. 8). The physical transport of highly perishable goods to often-distant markets often required business connections and unique logistical support that was unavailable outside of intermediaries (Bhuiyan et al. 2021; Inomata et al. 2020; Prabhu et al. 2019). “[T]he distance from the abalone fishing sites to the marketplace. This creates the need for middlemen" (Taridala et al. 2020, p. 2947). In Vietnam, middlemen contributed to governance and resource conservation by functioning as a kind of harvest tax, which would otherwise need to be managed by the government (Thủy et al. 2019). Intermediaries were described as “perform[ing] a fundamental supply service” (Bevilacqua et al. 2019, p. 333) and as “influential and crucial to the proper functioning of value chains" (Adetoyinbo and Otter 2021, p. 194), a role so important that it was recognized in FAO’s voluntary guidelines for SSF (González-Mon et al. 2019).

#### Impacts on resource management

One-fifth of the documents (n = 17) discussed intermediaries as impacting the physical environment around them, generally in relation to fish stocks or resource conservation. A few of these emphasized the positive role traders can have on the environment, for example by leveraging their Traditional Ecological Knowledge as “an important function for the conservation of biodiversity of fish species in the region” (Alfian et al. 2020, p. 393). Thủy et al. (2019) detailed how “middlemen with market power can help protect marine resources from depletion by implicitly ‘taxing’ the harvest” (p. 439), thus “contribut[ing] to conservation of the fish stocks” (p. 451), which for management efforts can “be a second-best policy option” (p. 452). Coronado et al. (2020) noted the potential for middlemen to “reduce pressure on resources in danger of being overfished” (p. 7) given their position as “key links between communities and alternative buyers” (p. 7).

In describing efforts to obtain Fair Trade certification, Doddema et al. (2020a) detailed how an NGO managed to gain traders’ support to implement traceability standards and the importance of traders’ buy-in for these measures to be implemented. They found that “despite the Fair Trade groups being formed and organized around them …, the Fair Trade requirements do not recognize their role or give recognition to the changes made by traders to facilitate the Fair Trade certification of the fishery,” thus complicating efforts to leverage traders to meet global standards (Doddema et al. 2020a, p. 12).

Most discussions of intermediaries’ impacts on the physical environment emphasized the destructive effects of their involvement. This was primarily linked to market incentives and the provision of destructive equipment. Both low and high prices paid to fishers were linked to overfishing, with the former as resulting in “increased extraction [as] the only way for fishers to earn enough income” (Jimenez et al. 2019, p. 8) or manage their debt to intermediaries (Drury O’Neill et al. 2019a, b; González-Mon et al. 2019) and the latter as driving “fishermen to catch every last crustacean entangled in gillnets” (Saputra 2020, p. 465). High market demand in the form of foreign and local intermediaries was also shown to drive overfished quotas (Pedroza-Gutiérrez and López-Rocha 2021) and the “illegal harvest of marine resources” (Patankar 2019, p. 2). Intermediaries’ supply of “destructive fishing gears (i.e., cyanide and bombs)” (Vandenberg et al. 2021, p. 76) and “small mesh size and monofilament gillnets” (Getahun et al. 2020, p. 330) was presented as a major problem with the potential to undercut restoration efforts. Additionally, the mere indifference of intermediaries to resource management had potential environmental implications (María Esther et al. 2021).

#### As vulnerable or struggling themselves

Just under 13% (n = 11) of documents included framings of intermediaries, most commonly women, as vulnerable or struggling actors in the supply chain. This is a contrast to the predominant framing of intermediaries as having greater influence in the supply chain than other actors (as described in Sect. "Dominance, power, and influence"). In Ghana, Appiah et al. (2021) found that “[t]he majority of the women fish processors-traders in the communities (56.8%) were classified as moderately vulnerable to livelihood insecurity, while 34.2% were classified as highly vulnerable, and only 9% were found within the low vulnerability category” (p. 1609), and that this vulnerability was across “almost all the indicators apart from ‘ownership of mobile phones’, indicating their low access to capital assets” (p. 1613). This vulnerability also linked to their marital status such as “the social stigma associated with widowed fish vendors,” who were “[s]ocially marginalized due to their marital status … [and] face challenges in accessing fish markets, loans or finding other types of employment” (Azmi et al. 2021, pp. 637, 631). In systems where wives typically traded their husbands’ catch, shocks such as the scarcity of specific species were shown to lead to “unemployment [that] eventually forced the women (ex-swimming crab processors) to work abroad. This decision to work outside the village created family conflict and led to divorces” (Saputra 2020, p. 468).

Women traders and processors were also viewed with suspicion from other traders and fishers in the supply chain. “The young women who engaged in night vending were spoken of (by other vending women and the village community at large) as the ones with sexual urges. ‘People say that young women go to Attingal market to buy ‘etthakka’ (banana, referring in double meaning both to the fruit and the male reproductive organ) and to have affairs with men’” (P. Aswathy and Kalpana 2019, p. 200). At a meeting between fishers and the fisheries department in Togo, some fisher organizations were “bothered to see women [processors] express themselves. ‘These women are going to spoil it all’, argued one of them who does not understand why women are allowed to speak at all” (Cele 2020, p. 147). The vulnerability of women traders was also seen to be as a consequence of other female traders, for example in the Lake Victoria region of East Africa where “women traders from the DRC manoeuvered themselves in positions from which they could manipulate the network through bribery and conniving to derive substantial capital gains from the kayabo trade, their Tanzanian counterparts however are excluded from the decision-making processes, access to fish resources, financial capital, and negotiation power” (Medard et al. 2019, p. 321). Intermediaries’ struggles were not always gender specific. Around the East African lakes of Victoria and Turkana, traders were more constrained by social ties than fishers and noted potential physical aggression by fishers should they attempt to recoup money from unfaithful fishers (Nunan et al. 2020), and cooperatives were described as under threat from both fishers and other traders’ payments to fishers (Getahun et al. 2020).

## Discussion & conclusion

### Predominant themes

The most recent literature, as represented by our subset, has framed intermediaries both positively and negatively. Though the negative framings demonstratively outweighed the positive, it is encouraging to see depicted the nuances of relationships between intermediaries and other actors. These nuances matter because intermediaries writ large are not a homogenous group. Globally, and even locally, they vary substantially by gender, ethnicity, scale of operation, kinship ties to fishers, etc. Broad labels such as ‘trader’ or ‘wholesaler’ obscure substantial diversity in how actors are positioned within seafood supply chains and the power relations they navigate. Within the same supply chain, some intermediaries may function as essential linchpins, while others may extract value as leeches, and still others may embody both roles simultaneously. Thus, these positive and negative framings and the narratives they inform are fundamental as they have the potential to transform supply chains towards sustainability and equity through more just and context-sensitive governance decisions.

Documents frequently framed intermediaries as bottlenecks (see Sect. "As bottlenecks"), limiting critical access to finance, equipment, and markets; sometimes this was said to be a strategy employed by intermediaries (see Sect. "Exploitation and manipulation"), and at other times it also stemmed from the unique logistical challenges of SSF supply chains (see Sect. "As bottlenecks"). This resonates with (Purcell et al. 2017), who illustrated global seafood supply chains as hourglass shaped, with intermediaries positioned at the narrowest point. In our review, framing intermediaries as bottlenecks (see Sect. "As bottlenecks") not only highlighted the binding relationships and dependency of others on intermediaries (see Sect. "Binding or limiting the options of others") but also reinforced the asymmetrical and inequitable nature of intermediary-related interactions (see Sects. "As bottlenecks" and "Contributing to the inequity or inequality of others") that sustain their relational power (see Sect. "Dominance, power, and influence"). This power enables the exploitation of other actors (even other intermediaries), particularly in relation to gatekeeping or withholding access to critical services and support, dictating prices, and guaranteeing a steady supply of fish to intermediaries (see Sect. "Exploitation and manipulation"). These interactions between socio-economic factors and market prices are markedly complex in heterogeneous fisheries contexts, warranting additional examination (Wamukota et al. 2014). At times labeled as corrupt, manipulating, or monopolistic middlemen (see Sects. "Exploitation and manipulation"–"As bottlenecks"), this narrative of intermediaries has underpinned calls for them to be replaced in or eliminated from supply chains (see Sect. "Elimination or replacement as an improvement or goal") in order to improve their equitable functioning, though this has proven to be difficult. However, despite this narrative of intermediaries as leeches, these actors have persisted. No doubt linked to the reinforcing nature of power, inequality, dependency, and exploitation, this begs the question of what essential roles they also play to maintain their influential positions in supply chains (Ferdous and Nunan 2025; see Sect. "Necessary for the supply chain").

In contrast, positive framings of intermediaries emphasized their enabling, supportive, or buffering roles. Though in our review, these framings were both less prevalent within the subset and more nuanced than the negative, they noted the perceived and inherent riskiness of SSF systems (see Sects. "Binding or limiting the options of others" and "Necessary for the supply chain"), which may be a reason why other, more formal institutions such as banks are not engaged (Fabinyi et al. 2018; Idrus 2009). The non-involvement of other institutions positions intermediaries as linchpins within the supply chain, facilitating the essential links between small fishers and larger markets (see Sect. "Necessary for the supply chain"). In addition to facilitating critical market linkages, they were described as providing forms of social protection (e.g., credit, informal insurance, social support) (see Sect. "Social protection and social insurance"), or as improving the supply chain or situation of other actors through the, otherwise unavailable, provision of infrastructure, knowledge, or market access (see Sect. "Improving the supply chain or situation of other actors"). We note that some of these positive aspects have been raised previously in the literature: the critical market linkages (Bailey et al. 2016), the social protection aspect (Ferrol-Schulte et al. 2014; Ferse et al. 2012; Idrus 2009), and the buffering role of credit (Firth 1946; Platteau and Abraham 1987).

Nuanced framings offered a more complex picture, highlighting how intermediaries can concurrently enable and constrain different outcomes. In line with Ruddle’s (2011) research on “the flexible and multiple economic and social services that [intermediaries] perform in fishing communities” (p. 230), multiple documents highlighted that rural livelihoods would suffer without intermediaries, framing them as necessary (see Sects. "Social protection and social insurance" and "Necessary for the supply chain"). However, others noted that this didn’t always mean that the terms of their engagement were favorable for the fishers; in fact, it could leave them further in debt. In some contexts, intermediaries “manipulated” the supply chain to situate themselves as necessary (see Sect. "Exploitation and manipulation"), whereas in other situations they seemed to fill a pre-existing gap (see Sects. "Improving the supply chain or situation of other actors"–"Necessary for the supply chain"). Demonstrating a pattern of wishful thinking, intermediary institutions such as cooperatives were proposed as more equitable alternatives to conventional middlemen (Bavinck et al. 2018; see Sect. "Elimination or replacement as an improvement or goal"). Some were successful in achieving more equity for fishers, while others failed in their attempts to replace or circumvent middlemen or merely perpetuated longstanding asymmetries (see Sect. "As bottlenecks"). In some cases, intermediaries were described as wielding influence in ways that empowered themselves at the expense of other supply chain actors, including other intermediaries (see Sects. "Dominance, power, and influence"–"As bottlenecks"). Other accounts emphasized intermediaries as vulnerable or struggling themselves, embedded within volatile markets and precarious livelihoods (see Sect. "As vulnerable or struggling themselves"). Other documents pointed to their impacts on the physical environment, ranging from exacerbating overfishing to facilitating more sustainable practices (see Sect. "Impacts on resource management"), as previously noted in Crona et al. (2010), Ferse et al. (2012), Ferrol-Schulte et al. (2014), and Nurdin and Grydehøj (2014). Across most of the sub-themes described above, the theme of dominance, power, and influence frequently overlaps; this is because when paying attention to the nuance of what is being discussed, one sees that an intermediary’s influence can support positive transformation of supply chains, but also reinforce inequities within those very supply chains (see Sect. "Dominance, power, and influence").

### Discussion of methods

Scoping reviews are increasingly being used in the social sciences to distill the complexity of burgeoning literature (Logan et al. 2024). By following an established protocol and comprehensively tested search strategy, a systematic scoping review provides a transparent, replicable, and meticulous approach to collecting and synthesizing a large body of literature. By having more than one reviewer, the risk of individual bias is minimized, rendering it particularly valuable for qualitative fields that are interpretively rich but sometimes criticized for being methodologically opaque. The field of evidence synthesis has recently established frameworks and guidelines to effectively conduct scoping and systematic reviews (Arksey and O’Malley 2005; Tricco et al. 2018). However, adherence to these guidelines remains inconsistent in fields such as social sciences. Through this study, we aimed to provide a practical demonstration of scoping review methods for marine social scientists. The effective adoption of these research methods could greatly assist the marine social sciences in distilling broad and complex issues into streamlined and practical avenues for additional research.

As with all research, the framing of the actual research question influences the research outcomes. This scoping review allowed us to understand the extent to which intermediaries have been examined in the wider literature, particularly in the most recent years. The broad scope highlighted the growing interest in these actors, while the narrowed analytical focus of this review permitted us to capture the nuances and contradictions of how they have been normatively framed in the literature. By methodically reviewing such an expansive body of literature, we were able to visualize the geographical spread of the research conducted on this theme since 1951, allowing us to note the frequency of intermediary research in Indonesia (see Online Resource 1). Noting how widespread the discussion of intermediaries was in regards to their dominance, power, and influence, our qualitative analysis of the subset enabled us to delve into the nuances of how these attributes shape a wide range of arguments, such as how their power can be used for good (e.g., social protection) or bad (e.g., as binding or limiting).

However, scoping reviews have limitations. The process can be quite labor intensive, as it requires careful coordination, multiple screening rounds, iterative coding, and thus may be challenging for small teams with tight deadlines. For example, the majority of this scoping review has focused on the most recent subset of literature, and further analysis could be done on the full set of 380 documents and/or of the literature published since this search. Handling and screening the large volumes of research articles generated by systematic searches also involves organizational skills or review management software to keep track of which articles have been reviewed by which reviewers. We also recommend clearly defining the scope and search strategy to keep the task manageable. Being firm about inclusion criteria can help sort through large volumes of articles and exclude those which only superficially engage with the topic of interest.

### Future research & conclusion

Previous scholarly research has documented the diversity of roles of seafood market intermediaries in supply chains; however, this paper’s contribution is to methodically review and summarize the normative framings underlying their depictions in recent literature. Through this scoping review, the contrasting framings of intermediaries as leeches and/or linchpins becomes apparent. Together, these normative framings contribute to narratives that “inform general and sectoral debates over development,” and by doing so, “inform and give power to interpretations of change and policy in fisheries” (Johnson 2006, p. 751). This influence is visible in suggested or attempted interventions across the review. Multiple studies directly link asymmetrical and exploitative relationships with intermediaries as a cause of structural poverty and an obstacle to escape it (e.g., Darwis and Zulkarnain 2020; Kinseng et al. 2019; Nurlaili et al. 2019; Pomeroy et al. 2020; see Sects. "Exploitation and manipulation" and "Contributing to the inequity or inequality of others"). This and other negative framings of intermediaries have led to interventions aiming to eliminate, circumvent, or replace them. Suggested interventions have included: cooperatives (e.g., Ertör-Akyazi 2020; Fitrianggraeni 2019; Hamzah and Nurdin 2021; see Sect. "Elimination or replacement as an improvement or goal"), government-supported microfinance and subsidies (e.g., Anwar et al. 2019; Jueseah et al. 2020; Kimani et al. 2020a; see Sect. "Elimination or replacement as an improvement or goal"), and the implementation of Fair Trade standards and EU IUU regulations (Doddema et al. 2020a; see Sect. "Impacts on resource management"). In contrast to these attempts to circumvent intermediaries, FAO’s 2015 voluntary guidelines for SSF with regard to food security and poverty eradication has recognized the necessity of intermediaries for SSF supply chain functioning and suggested their inclusion in decision-making processes (González-Mon et al. 2019; see Sect. "Necessary for the supply chain").

Rather than binary framings of intermediaries as good or bad for supply chains, more focus is needed on their positive and nuanced roles. Previous cases studied where intermediaries were framed solely negatively should be re-examined to see if intermediaries’ roles are more nuanced. While some research has posited that the key impediment to equity is the existence of monopolies, attempts to overcome intermediary-maintained monopolies (whether by means of breaking up or eliminating them) should be sensitive to the socio-economic roles these actors fulfill that reproduce these structures. Research should also explore the boundaries between exploitation and equitable business practices given the unpredictable nature of SSF (i.e., in business terms, high-risk). For instance, what *would* be a reasonable profit margin that equitably reflects the costs of buffering risk? Future research should incorporate a gender-sensitive approach to help raise the underlying complex social relationships to inform both policy recommendations and robust science (Hapke 2001; Kaminski et al. 2020; Overå, 1993). Additionally, research should examine the social-ecological roles intermediaries can play in fostering equity, food and nutrition security, and fisheries governance reform; a geographical disaggregation could enhance the potential for effective interventions. As demonstrated by this scoping review, intermediaries are key actors in supply chains. Due recognition of the important functions they fulfill in supply chains can inform interventions aimed at equitable outcomes for everyone.

## Supplementary Information

Below is the link to the electronic supplementary material.

Supplementary file1 (PDF 604 KB)

Supplementary file2 (CSV 590 KB)

## Data Availability

A dataset of all the documents included in the final screening stage is provided in the Supplementary Information.
