# Supplementary material for: Leeches and linchpins: a scoping review of seafood market intermediaries in small-scale fishery supply chains
Source: Marit Stud. 2026 Oct 19;25(4):64. doi: 10.1007/s40152-026-00516-6 (PMC13680514; doi:10.1007/s40152-026-00516-6)
Supplement: Supplementary file 1 [file 40152_2026_516_MOESM1_ESM.pdf]

Online Resource 1

Supplemental Table 1: Inclusion and exclusion criteria used for the full text screening stage (n = 901 documents). By providing clear criteria by which documents could qualify for inclusion or exclusion, this table served as a guide for us to remain consistent and systematic throughout this screening stage. Note that not all inclusion or exclusion criteria needed to be present to make a screening decision.

INCLUSION	EXCLUSION
**SSF** (Marine + Freshwater)<br><br>**Peer-review, grey literature** (reports, policy brief), conference paper (with extractable data)<br><br>**Seafood intermediary** included and role /perception of described<br><br>**Gendered** aspects of middlemen<br><br>Discusses **normative framing** of intermediaries (good/ bad/ other)<br><br>Discusses **potential for intermediaries to transform systems** to be more: sustainable, just, or food secure (food safety/ utilization not included here).<br><br>Discussion of **pricing** which reflects on power/influence of the relationships on the price (price taker/giver/equiter). Document discusses the how or why of prices as opposed to only reporting the prices with no additional analysis.	**High level**- Industrial fisheries/ too high level<br><br>**No SSF**- No discussion of fish or SSF<br><br>**Abstract**- Conference abstract or poster (too preliminary or no extractable data)<br><br>**Producer-only**- Doesn't discuss intermediaries or relationships with intermediaries<br><br>**Consumer-only**- Doesn't discuss intermediaries or relationships with intermediaries<br><br>**Quotas**- Tradeable fish stocks/ ITQs- incl boat sales/ buyback schemes<br><br>**Aquaculture**- Aquaculture Only<br><br>**Plants**- Aquatic plant discussion only<br><br>**Fish4Sex**- very HIV/AIDS transmission-focused; no discussion of fish prices, negotiations, power<br><br>**Seafood fraud**- document ONLY deals with **incidental** mislabeling as opposed to **intentional** seafood fraud<br><br>**Safety- consumer**- food safety- document ONLY discusses consumer utilization (i.e., the body's absorption of nutrients or lack thereof)

	**Safety- worker**- document ONLY discusses occupational hazards for fish smokers and other fish workers<br><br>**Slavery/ forced labor**- typically only applicable to industrial fisheries, production or marketing are both out of our realm<br><br>**Modeling**- Game theory & agent-based modeling- with NO mention of (any non-theoretical) power dynamics that underpin/ structure the model. I.e., **no real-world case data underpinning the model**<br><br>**Ancient world**- doesn't address intermediary relationships or power dynamics<br><br>**Angler**- Anglers/ recreational fishing<br><br>**Non-food**- Non-human-food fish products<br><br>**Gear**- Allied fishing activities only (boat making, gear, etc)<br><br>**Non-market co-op**- Co-op description does not include marketing aspect<br><br>**Price only**- Discussion of pricing doesn't include power/ influence of the relationships on the price (price taker/ maker). Document only reports/ lists fish prices with no analysis or rationale for price setting<br><br>**Superficial**- References to/ mentions of intermediaries are superficial with no extractable use

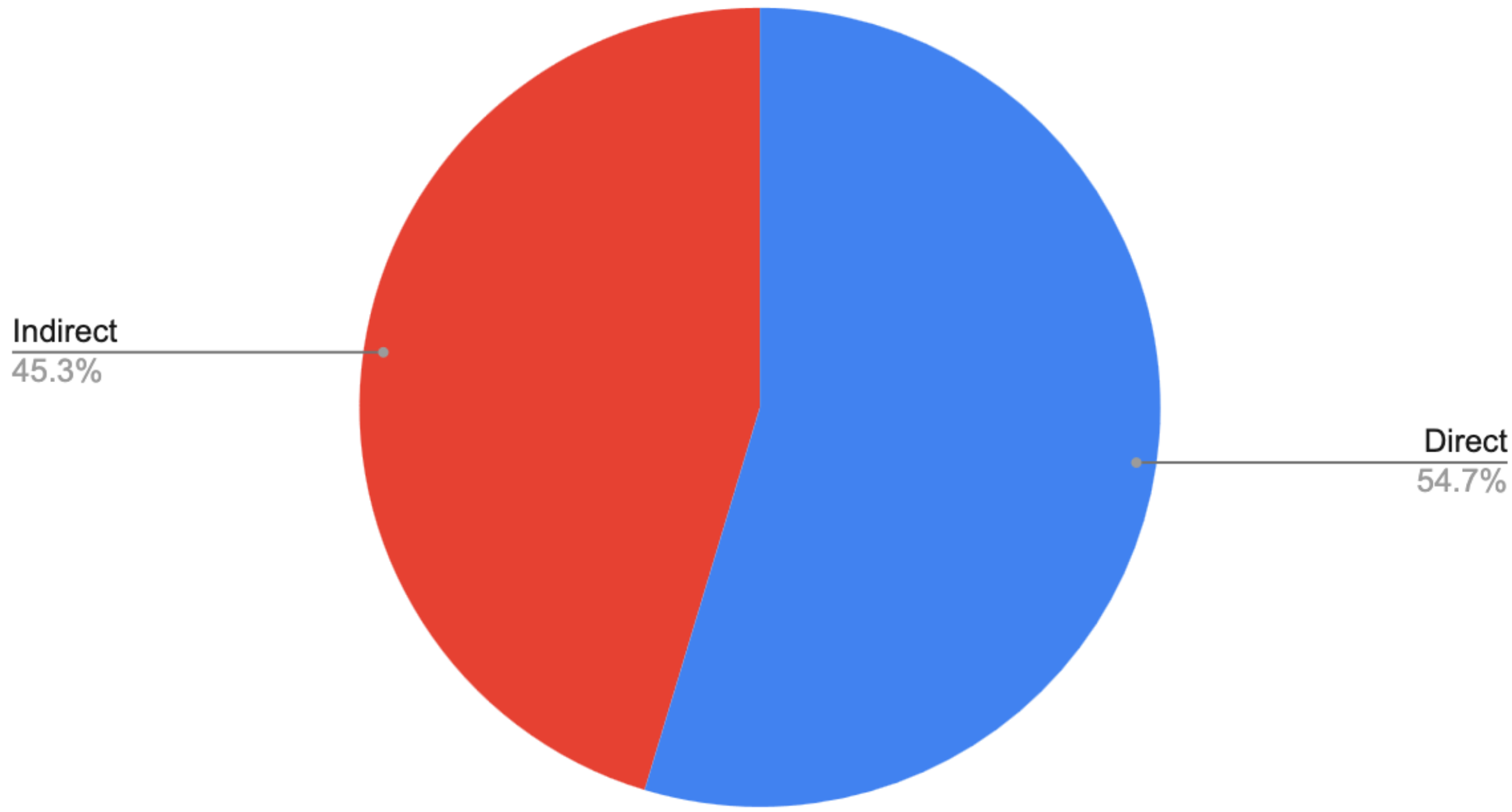

Supplemental Fig. 1 Proportion of documents (n = 86) that focused on seafood market intermediaries directly vs only indirectly mentioning them in the overall document. Indirect mentions involved documents focused on other aspects or aspects of the SSF.

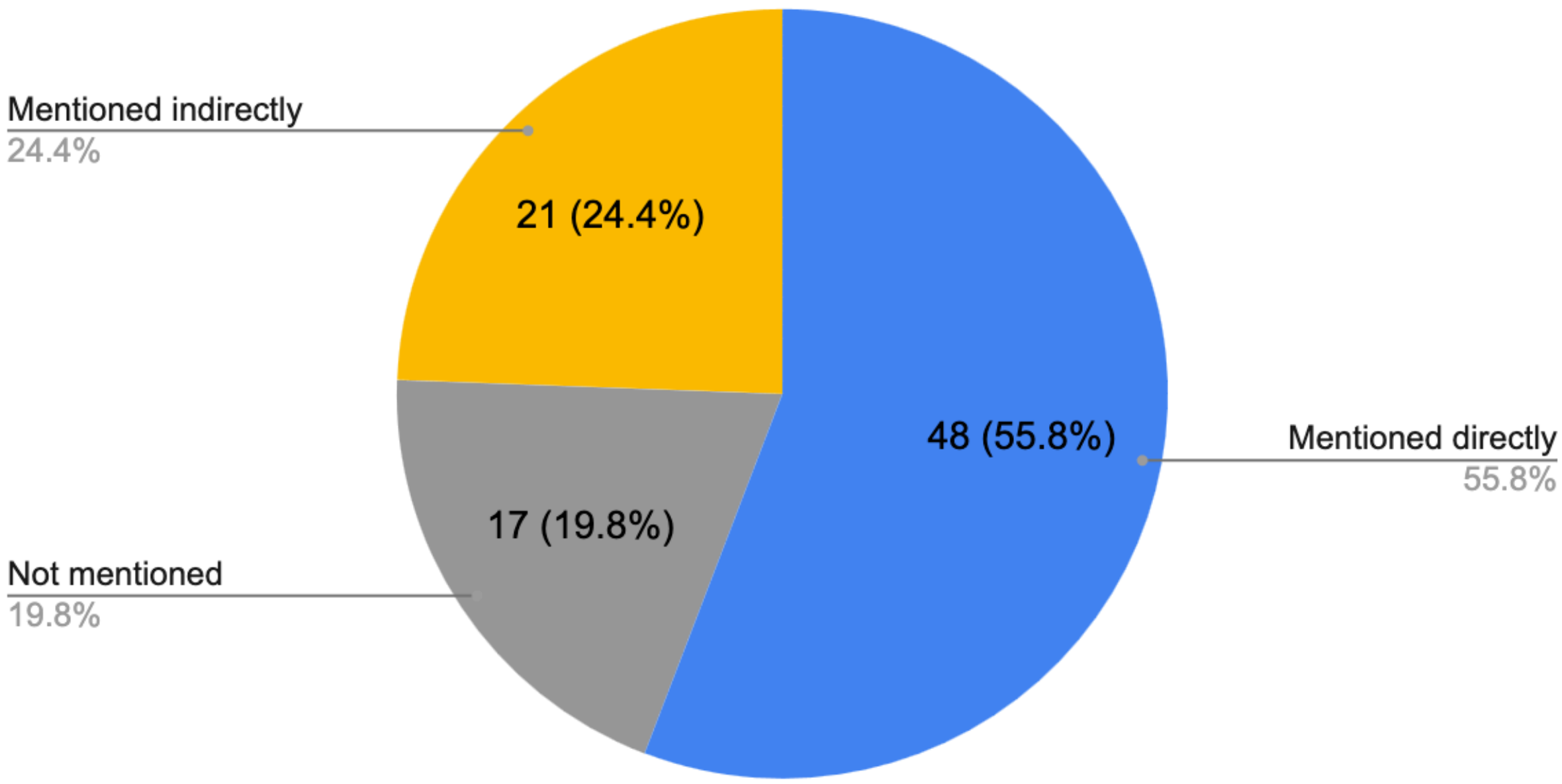

Supplemental Fig. 2 Proportion of documents (n = 86) that mention the term power directly (55.8%), indirectly (24.4%), or not at all (19.8%). Indirect mentions involved allusions to power or power dynamics.

Supplemental Fig. 3 Proportion of documents (n=28) focused on different regions within

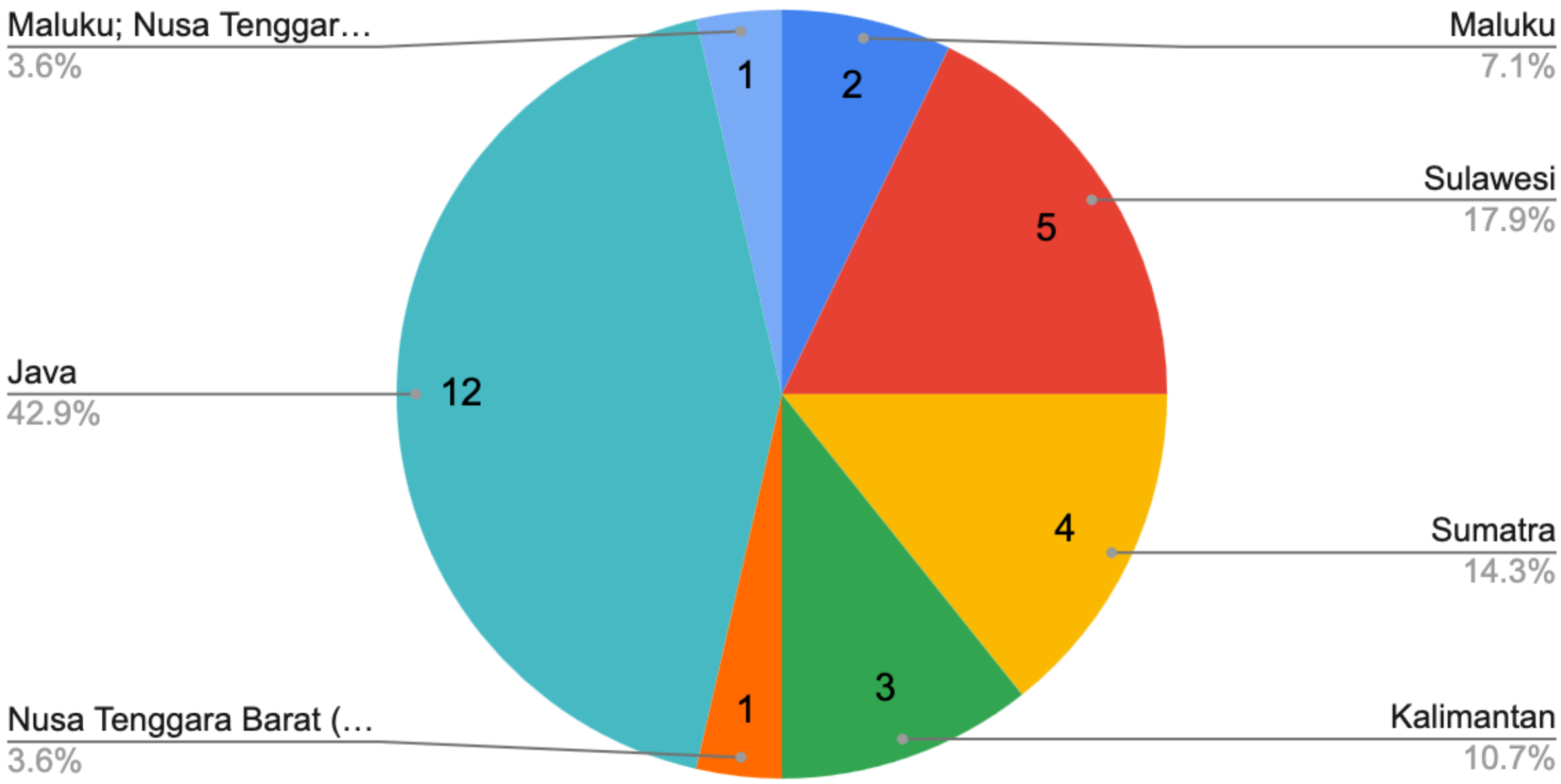

Indonesia. Of the 28 articles focused on Indonesia, almost half (42.9%) were situated on the heavily populated island of Java. The next most commonly studied regions were Sulawest (17.9%) and Sumatra (12.3%).

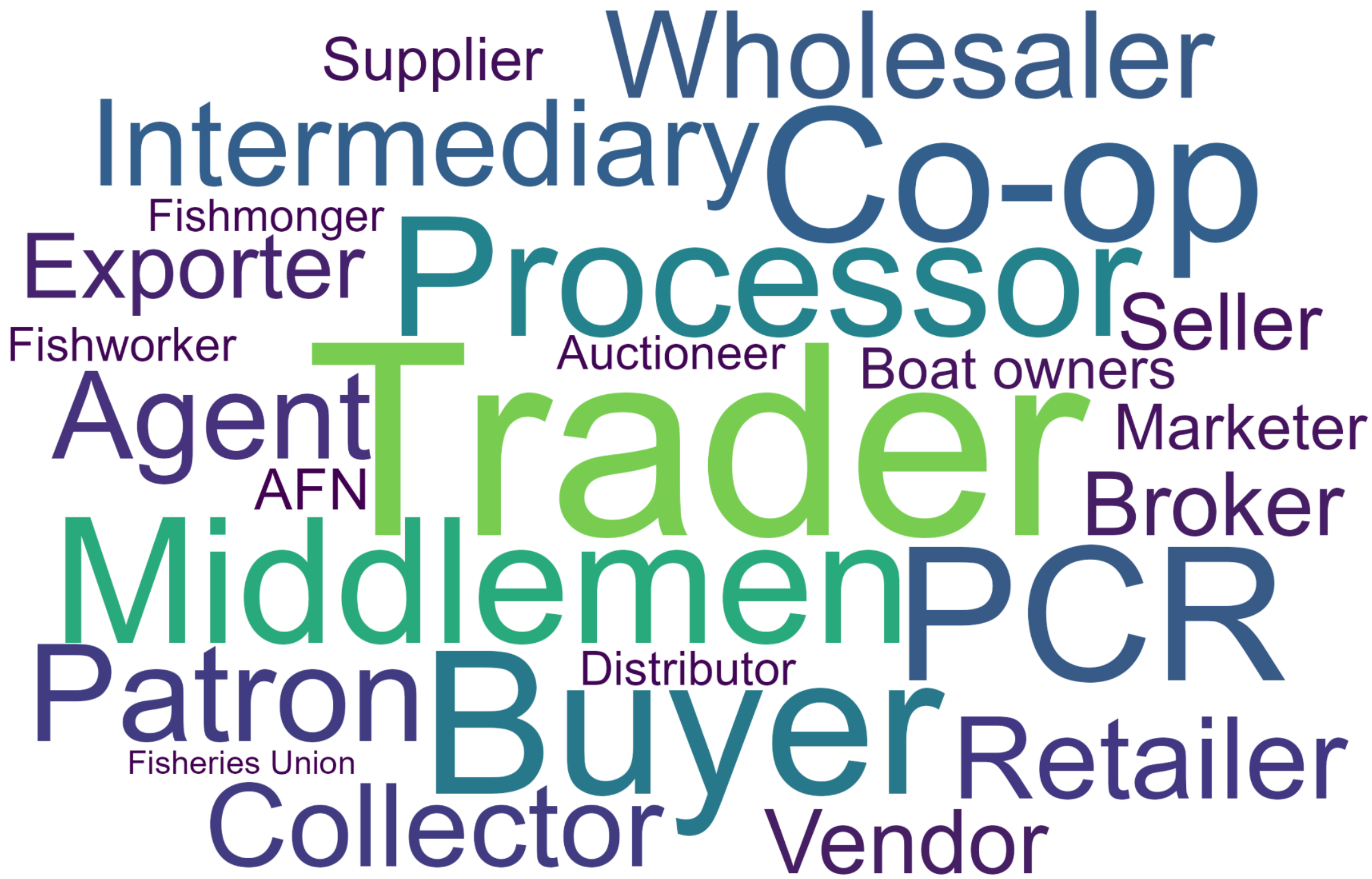

Supplemental Fig. 4 Word cloud depicting frequency of terms used to describe seafood market intermediaries in the subset (n = 86). Single instances of terms are not displayed. (AFN = Alternative Fisheries Network; PCR = Patron-Client Relationships)
